# Supplementary material for: Response of blueberry photosynthetic physiology to light intensity during different stages of fruit development
Source: PLoS One. 2024 Sep 25;19(9):e0310252. doi: 10.1371/journal.pone.0310252 (PMC11423972; doi:10.1371/journal.pone.0310252)
Supplement: Attachment — Submitted filename: Responses to reviewers.docx [file pone.0310252.s002.docx]

Responses to reviewer 1:

1. the information on shaping and pruning has been removed.

2. regarding the suggestion on density control, the conclusion has been revised and pointed out that plants should be reasonably spaced to avoid shading each other.

3. Site selection is recommended to choose sunny and shade-free areas. (Modified conclusions have been noted)

4. The recommendation on Table 1 has been modified.

5. Concerning the fruit quality parameters lack of consideration in this study, our group will further improve it in the future.

6. The article has pointed out 3 sets of replications for each treatment, where each replication is 10 plants, i.e.: 30 blueberry plants for each treatment.

7. The section (line 93) has been amended.

8. The article has indicated that the variety is called 'O'Neill'.

9. the statistical analysis has been revised.

10. All abbreviations in the revised graph have been explained in the title.

11. fonts have been adjusted to improve graphic quality.

Thank you for your valuable suggestions!

Responses to reviewer 2:

1 Abstract

Has been improved to a more concise summary.

2 Introduction

Repetitive information about the introduction has been fixed. The setup of the light intensity range in this study covers a wide range of light intensities and can be illustrative. Few studies have been conducted specifically on the photosynthetic response of blueberries to light intensity, so the context is not as focused.

3 Materials and Methods

The formulas of the revised article on photosynthetic parameters have provided the background. Relative light intensity was controlled with shade nets of different densities. The method of ultra-low temperature storage had less effect on chlorophyll content. Measurements of photosynthetic parameters were determined with a photosynthesizer at the experimental base.

4 Discussion

The discussion mentions that in previous studies: low light intensity has both negative and positive effects on chlorophyll synthesis, while the article points out that in this study low light intensity had a positive effect on chlorophyll synthesis. The question about stomatal conductance has been revised. The reason behind the changes in photosynthetic parameters and photosynthetic pigments under different light intensities has been added.

5 Conclusion

Based on the suggested modifications, the conclusion has indicated the optimum light conditions for different stages of fruit development.

Thank you for your valuable suggestions on this article!
